# Influence of Ethnicity on the Accuracy of Non-Invasive Scores Predicting Non-Alcoholic Fatty Liver Disease

**DOI:** 10.1371/journal.pone.0160526

**Published:** 2016-08-31

**Authors:** Ming-Feng Xia, Hannele Yki-Järvinen, Hua Bian, Huan-Dong Lin, Hong-Mei Yan, Xin-Xia Chang, You Zhou, Xin Gao

**Affiliations:** 1 Department of Endocrinology and Metabolism, Zhongshan Hospital, Fudan University, Shanghai, China; 2 Institute of Chronic Metabolic Diseases, Fudan Unversity, Shanghai, China; 3 Department of Medicine, University of Helsinki, Helsinki, Finland; 4 Minerva Foundation Institute for Medical Research, Helsinki, Finland; Taipei Veterans General Hospital, TAIWAN

## Abstract

**Objectives:**

Presence of non-alcoholic fatty liver disease (NAFLD) can predict risks for diabetes, cardiovascular disease and advanced liver disease in the general population. We aimed to establish a non-invasive score for prediction of NAFLD in Han Chinese, the largest ethnic group in the world, and detect whether ethnicity influences the accuracy of such a score.

**Methods:**

Liver fat content (LFAT) was measured by quantitative ultrasound in 3548 subjects in the Shanghai Changfeng Community and a Chinese score was created using multivariate logistic regression analyses. This new score was internally validated in Chinese and externally in Finns. Its diagnostic performance was compared to the NAFLD liver fat score, fatty liver index (FLI) and hepatic steatosis index (HSI) developed in Finns, Italians and Koreans. We also analyzed how obesity related to LFAT measured by ^1^H-MRS in 79 Finns and 118 Chinese with type 2 diabetes (T2D).

**Results:**

The metabolic syndrome and T2D, fasting serum insulin, body mass index (BMI) and AST/ALT ratio were independent predictors of NAFLD in Chinese. The AUROC in the Chinese validation cohort was 0.76 (0.73–0.78) and in Finns 0.73 (0.68–0.78) (p<0.0001). 43%, 27%, 32% and 42% of Chinese had NAFLD when determined by the Chinese score, NAFLD liver fat score (p<0.001 vs. Chinese score), FLI (p<0.001) and HSI (NS). For any given BMI and waist circumference, the Chinese had a markedly higher LFAT than the Finns.

**Conclusion:**

The predictors of NAFLD in Han Chinese are as in Europids but the Chinese have more LFAT for any given degree of obesity than Europids. Ethnicity needs to be considered when NAFLD is predicted using risk scores.

## Introduction

Non-alcoholic fatty liver disease (NAFLD) is defined as steatosis, which is not due to alcohol or other known causes of liver disease [1]. NAFLD, a hepatic manifestation of the metabolic syndrome (MetS) [2], is the leading cause of chronic liver disease [3].NAFLD predicts both type 2 diabetes (T2D), cardiovascular disease and advanced liver disease independent of obesity [4]. Thus, identification of individuals with NAFLD is of interest. However, according to an American guideline, “Screening for NAFLD in adults attending primary care clinics or high-risk groups attending diabetes or obesity clinics is not advised at this time due to uncertainties surrounding diagnostic tests” [1]. The statement implies that the accuracy of diagnostic tests for NAFLD should be improved.

Ultrasound (US) is the most commonly used tool to diagnose NAFLD [5]. However, this technique has low sensitivity of detecting mild steatosis in obese subjects [6]. We have recently shown that the accuracy of US can be improved by application of an external phantom and reference data for liver fat content (LFAT) measured by proton magnetic resonance spectroscopy (^1^H-MRS) [7]. Indeed, a guideline by the Chinese Study Group of Liver and Metabolism recommended “US-based screening for NAFLD in high-risk adults, especially those who attend diabetes or obesity clinics” [8]. As the prevalence of NAFLD is increasing globally, there is a need to discover even simpler tools than US for diagnosis of NAFLD.

Several scores have been developed for estimating LFAT in different ethnic groups. These include the NAFLD liver fat score developed in Finns (based on MetS, T2D, insulin, AST, AST/ALT) [9], the fatty liver index (FLI) in Italians body mass index (BMI), waist circumference, triglycerides (TG), γ-glutamyltransferase [10], the Steatotest^R^ developed in French (12 variables in an undisclosed formula) [11],and the hepatic steatosis index (HSI) developed in Koreans (ALT/AST, BMI,T2D) [12].

Although Han Chinese are the largest ethnic group in the world [13], no score has been developed and validated for predicting NAFLD in this ethnic group. The Chinese with NAFLD appear to be leaner than Caucasoids [14]. However, no study has compared how obesity, in Chinese compared to other ethnic groups, correlates with LFAT.

In the present study, we i) developed a non-invasive diagnostic score and validated it internally and externally for screening NAFLD in Han Chinese; ii) compared the performance of the Chinese score with the NAFLD liver fat score, FLI and the HSI in Chinese and Finns, and iii) compared the relationship between obesity and LFAT measured with ^1^H-MRS between Chinese and Finns.

## Subjects and Methods

A total of 4576 middle-aged and elderly Chinese community subjects were consecutively enrolled from May 2010 to June 2012 [15], and 1028 participants were excluded due to known viral hepatitis B (n = 163), viral hepatitis C (n = 3), excessive alcohol consumption (≥140g per week for men and ≥70g per week for women) [16] (n = 627) and use of hypoglycemic drugs or insulin (n = 235). This left 3548 subjects (1249 males and 2299 females) to be included in the final analysis. The Chinese participants were randomly divided into estimation (n = 2365) and validation (n = 1183) groups to build and internally validate the Chinese prediction score. Data of 572 eligible Finnish participants with varying degrees of NAFLD measured by ^1^H-MRS were used as an external validation cohort. The inclusion criteria for these subjects are: (1) age 18–75 years; (2) no known acute or chronic disease except for obesity or type 2 diabetes based on medical history, physical examination, and standard laboratory tests; and (3) alcohol consumption less than 140g per week for men and 70g per week for women. The subjects with incomplete of data or use of hypoglycemic drugs were excluded. The characteristics of part of this cohort have been previously described [9]. The flowchart of the study population was shown in S1 Fig.

In 119 of the 3548 Chinese participants, LFAT was also examined by^1^H-MRS. To compare the relationship between obesity and LFAT between Chinese and Finns, we analyzed data from previously studied 119 Chinese and from 79 Finnish patients with type 2 diabetes, in whom i) obesity was measured using BMI and waist circumference, ii) LFAT was measured by an identical technique (^1^H-MRS) and iii) blood samples were taken after an overnight fast for measurement of glucose, insulin and lipids, liver function tests and other measurements as described [9]. The subjects had no history or biochemical evidence of hepatitis B or C or of excessive alcohol consumption (≥20 g/day for men and ≥10g/day for women). They had no disease other than obesity or type 2 diabetes, did not use hypoglycaemic drugs or insulin, anti-hypertensives or other drugs possibly influencing glucose metabolism (S1 Table).

The study was approved by the Research Ethics Committees of the Shanghai Health Bureau and the University Central Hospital of Helsinki. Each participant provided written informed consent.

### Diagnosis of liver steatosis

LFAT was measured in 3548 Chinese community participants by a quantitative validated US method [7] and in 572 Finnish participants using ^1^H-MRS [9].

Trained ultrasonographists who were unaware of clinical data performed US examinations. US images were captured using a GE logiq P5 scanner (GE Healthcare, Milwaukee, WI, USA), analyzed using a NIH image software (ImageJ 1.41o, National Institutes of Health, Bethesda, MD) and standardized using a tissue-mimicking phantom (Model 057; Computerized Imaging Reference Systems, Norfolk, VA). LFAT was calculated using the equation: LFAT (%) = 62.592 * standardized US hepatic/renal ratio + 168.076*standardized US hepatic attenuation rate—27.863 [7].

### ^1^H-MRS measurements

^1^H-MRS were performed on 1.5-T magnetic resonance scanners manufactured by Siemens (Erlangen, Germany), and the intensity difference from various acquisition parameters and localization techniques was normalized as described in our previous work [9,17]. NAFLD was defined as LFAT≥5.56% [18].

### Other measurements

Body height and weight were measured without shoes and outer clothing for calculation of BMI. Waist circumference was measured using a soft tape midway between the lowest rib and the iliac crest in the standing position. In the Chinese participants, total cholesterol, HDL-cholesterol, TG were measured using oxidase method [19], and liver enzymes (ALT, AST) were measured by the UV lactate and malate dehydrogenase methods on a model 7600 automated bio-analyzer (Hitachi, Tokyo, Japan). The diagnostic kit was purchased from Donlim KELONG, Shanghai, China for ALT and AST, Roche for TG and total cholesterol, and Kyowa Medex for HDL cholesterol measurement. LDL cholesterol was calculated using the Friedewald equation. The fasting serum glucose concentration was measured using the glucose oxidase method. Fasting insulin concentrations were determined using an electrochemiluminescence immunoassay [17]. These measurements were also performed in Finns as described [9]. The reference values for fasting serum (fS) glucose were 3.9–6.0 mmol/l in Chinese and 4.0–6.0 mmol/L in Finns, for fS-HDL cholesterol >1.0 mmol/Lin men and >1.2 mmol/L in women in Chineseand Finns, fS-TG 0.6–1.7 mmol/L in Chinese and <1.7 mmol/L in Finns, ALT 9–50 U/L for men and 7–40 U/L for women in Chinese and ALT <50 U/L for men and <35 U/L for women in Finns, AST 15–40 U/L for men and 13–35 U/L for women in Chinese and AST 15–45 U/L for men and 15–35 U/L for women in Finns. These measurements were also performed in Finns as described [9].

### NAFLD liver fat score, FLI and HSI

Several prediction scores for diagnosing NAFLD have been developed in Finns (NAFLD liver fat score, ^1^H-MRS measured LFAT in 470 subjects [9]), Italians (FLIin the Dionysos study, US-based LFAT measurement in 496 subjects [10]), and Koreans (HSI, US-based study of 10724 subjects participating in a health check-up [12]) previously. In the present study, their diagnostic performance was further assessed in both the Chinese and Finnish participants.

### Statistical analysis

All statistical analyses were performed using SPSS software version 15.0 (SPSS, Chicago, IL) and R (http://www.r-project.org/). Data are shown as mean±SD. Univariate logistic regression analyses were used to calculate odds ratios (OR) and confidence intervals (CI) for NAFLD, and multivariate logistic regression analyses were used to establish the optimal model for prediction of NAFLD in the Chinese. Variables significantly associated with NAFLD in univariate logistic regression analyses were included in the analysis model. Receiver operating characteristic (ROC) curve analyses were used tovalidate the diagnostic performance of the new Chinese score in the internal Chinese validation group and external Finnish cohort, and compare its accuracy with NAFLD liver fat score, FLI and HSI for diagnosing NAFLD.The optimal cut-off value was determined using the Youden index [20]. The sensitivity, specificity, positive predictive values (PPV) and negative predictive values (NPV) were calculated as described [21], and the % of subjects with NAFLD using the 4 scores were compared by the Chi-squared test. Analysis of covariance (ANCOVA) was used to compare the slopes and intercepts of regression lines between Chinese and Finns. A two-tailed p-value of less than 0.05 was considered statistically significant.

## Results

### Baseline characteristics of the study population

A total of 3548subjects (1249 males and 2299 females) from Shanghai Changfeng community were enrolled and randomly divided into estimation (n = 2365) and validation (n = 1183) groups. The estimation and internal Chinese validation groups were comparable with respect to age, gender, BMI, LFAT, waist circumference, blood pressure, serum fasting glucose and insulin concentrations, lipids and liver enzymes (Table 1). The prevalence of NAFLD and T2D were 30% and 16% in the entire Chinese population. The Finnish group of 572 subjects was younger and had a significantly higher BMI, waist circumference, serum fasting glucose, insulin and liver enzyme concentrations, but their serum total cholesterol and LDL cholesterol were significantly lower than the Chinese (Table 1).

**Table 1:** Characteristics of the Chinese population and the Finnish external validation group.

	Chinese, all	Chinese, estimation group	Chinese, internal validation group	Finnish, external validation group	P Value	
					Estimation vs Validation group	All Chinese vs Finnish group
No.(% men)	3548(35.2)	2365(34.8)	1183(35.9)	572(43.7)	0.265	<0.001
Age (years)	63.3±9.8	63.1±9.8	63.5±10.0	45.0±8.0	0.239	<0.001
BMI (kg/m^2^)	24.2±3.4	24.2±3.4	24.2±3.3	34.9±8.8	0.523	<0.001
Waist (cm)	83.1±9.5	83.1±9.5	83.3±9.5	111.1±18.5	0.475	<0.001
Type 2 diabetes (%)	15.7	15.0	17.1	25.9	0.076	<0.001
Metabolic syndrome (%)	27.7	27.9	27.3	64.0	0.338	<0.001
Liver fat (%)	7.5±7.1	7.5±7.2	7.3±6.9	9.4±9.6	0.358	<0.001
NAFLD (%)	29.5	30.0	28.5	51.0	0.228	<0.001
fS-glucose (mmol/L)	5.49±1.28	5.48±1.29	5.50±1.27	6.33±2.64	0.775	<0.001
fS-triglycerides (mmol/L)	1.4(1.0–2.0)	1.4(1.0–2.0)	1.4(1.0–2.0)	1.4(1.0–1.9)	0.979	0.865
fS-HDL cholesterol (mmol/L)	1.44±0.37	1.44±0.38	1.43±0.36	1.29±0.41	0.168	<0.001
fS-LDL cholesterol (mmol/L)	2.91±0.79	2.91±0.79	2.91±0.80	2.84±0.93	0.901	0.055
fS-cholesterol (mmol/L)	5.10±0.93	5.10±0.93	5.09±0.94	4.84±1.10	0.700	<0.001
Systolic BP (mmHg)	135±19	135±19	135±19	134±17	0.841	0.236
Diastolic BP (mmHg)	76±10	76±10	76±10	84±11	0.265	<0.001
fS-insulin (mU/L)	9.56±7.20	9.51±6.96	9.66±7.65	12.28±8.37	0.579	<0.001
ALT (U/L)	16(12–21)	16(12–21)	16(12–22)	33(23–52)	0.431	<0.001
AST (U/L)	20(17–24)	20(17–24)	20(17–24)	30(24–40)	0.768	<0.001
GGT (U/L)	22(17–32)	22(17–32)	22(17–33)	32(20–58)	0.460	<0.001
AST/ALT ratio	1.3(1.0–1.6)	1.3(1.0–1.6)	1.3(1.0–1.6)	0.9(0.7–1.1)	0.557	<0.001

Data are in n (%), means ± SD or median (25th-75th percentile), as appropriate.

### Univariate analyses of determinants of NAFLD in Chinese

In univariate logistic regression analyses, NAFLD was associated with almost all studied parameters, including age, BMI, waist circumference, blood pressure, fasting serum glucose, triglycerides, HDL cholesterol, insulin, and liver enzymes, in all Chinese subjects and in the Finnish validation cohort (Table 2).

**Table 2:** Univariate logistic regression showing odds ratios and 95%CI for NAFLD in the Chinese and Finns.

	All subjects (N = 3548) OR (95%CI)	P value	Finns (N = 572) OR (95%CI)	P value
Gender (male)	0.971(0.831–1.134)	0.708	1.383(0.993–1.927)	0.055
Age (years)	0.989(0.981–0.997)	0.005	1.024(1.011–1.038)	0.009
BMI (kg/m^2^)	1.306(1.270–1.342)	<0.001	1.055(1.025–1.087)	<0.001
Waist (cm)	1.087(1.077–1.097)	<0.001	1.017(1.007–1.026)	0.001
Type 2 diabetes (%)	1.647(1.494–1.815)	<0.001	1.864(1.501–2.313)	<0.001
Metabolic syndrome (%)	3.674(3.123–4.323)	<0.001	4.364(2.790–6.825)	<0.001
fS-Glucose (mmol/L)	1.375(1.287–1.469)	<0.001	1.865(1.500–2.320)	<0.001
fS-triglycerides (mmol/L)	1.691(1.558–1.835)	<0.001	1.967(1.491–2.594)	<0.001
fS-HDL cholesterol (mmol/L)	0.266(0.212–0.333)	<0.001	0.167(0.090–0.311)	<0.001
fS-LDL cholesterol (mmol/L)	1.069(0.974–1.174)	0.159	1.248(0.988–1.576)	0.063
fS-cholesterol (mmol/L)	1.158(1.070–1.254)	0.165	1.120(0.916–1.369)	0.269
Systolic BP (mmHg)	1.011(1.007–1.015)	<0.001	1.017(1.004–1.031)	0.011
Diastolic BP (mmHg)	1.037(1.029–1.045)	<0.001	1.040(1.018–1.063)	<0.001
fS-insulin (mU/L)	1.147(1.129–1.166)	<0.001	1.117(1.076–1.159)	<0.001
ALT (U/L)	1.040(1.033–1.048)	<0.001	1.033(1.023–1.044)	<0.001
AST (U/L)	1.024(1.014–1.034)	<0.001	1.051(1.033–1.068)	<0.001
GGT (U/L)	1.004(1.002–1.007)	0.001	1.003(1.000–1.005)	0.041
AST/ALT ratio	0.233(0.190–0.285)	<0.001	0.173(0.092–0.324)	<0.001

OR, odds ratio; CI, confidence interval.

### Multivariate logistic regression analyses

In a multivariate logistic regression model, which included variables significantly associated with NAFLD in the univariate analyses (Table 2), the following variables independently predicted NAFLD: MetS, T2D, fS-insulin, AST/ALT and BMI (Table 3). Using this model, the risk score for NAFLD in the Chinese estimation cohort was as follows:

Chinese NAFLD score = -4.632 + 0.303 * MetS + 0.157*T2D (yes = 2/no = 0) +0.078* fS-insulin (mU/L) + 0.168*BMI (kg/m^2^) -0.879*AST/ALT

**Table 3:** Multivariate logistic regression model to predict NAFLD in Chinese estimation group.

	B	Standard error	P value	Odds ratio (95% CI)
Metabolic syndrome	0.303	0.130	0.020	1.354(1.048–1.748)
Type 2 Diabetes	0.157	0.073	0.032	1.170(1.013–1.351)
fS-insulin (mU/L)	0.078	0.012	<0.001	1.082(1.057–1.107)
AST/ALT	-0.763	0.132	<0.001	0.466(0.360–0.604)
BMI (kg/m^2^)	0.168	0.021	<0.001	1.183(1.135–1.233)
Constant	-4.632			

The variables entered the multivariate regression model include: BMI, waist circumference, T2DM, MetS, TG, HDL-c, TC, SBP, Insulin, ALT, AST, AST/ALT

### Diagnostic performance of the Chinese NAFLD score

**Fig 1:**
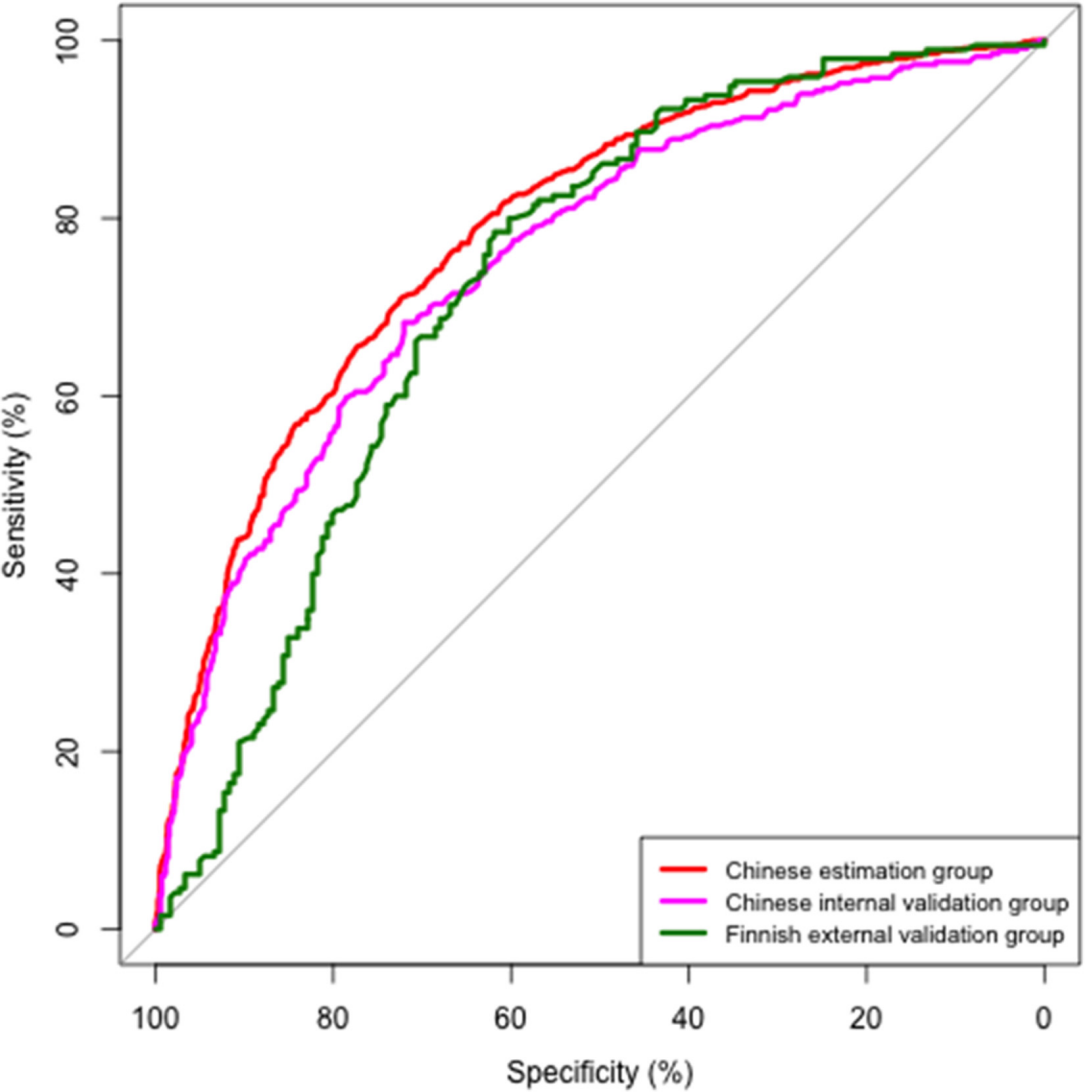
ROC-curves of the Chinese NAFLD score to predict NAFLD in the estimation group, the internal Chinese validation group and the Finnish external validation group. In the estimation group, AUROC = 0.79 (0.77, 0.81),cut-off = -0.79, Specificity = 0.72, Sensitivity = 0.71. In the internal Chinese validation group, AUROC = 0.76 (0.73, 0.78), cut-off = -0.79, Specificity = 0.71, Sensitivity = 0.69. In the Finnish external validation group, AUROC = 0.74 (0.69, 0.80), cut-off = 0.68, Specificity = 0.62, Sensitivity = 0.79.

In the estimation group, the area under the ROC curve (AUROC) was 0.79(0.77–0.81). By applying the Youden index to determine the optimal cut-off values for NAFLD, values greater than -0.79 predicted NAFLD with a sensitivity of 71% and specificity of 72% (Fig 1).

In the internal Chinese validation cohort, the AUROC of the Chinese NAFLD score was 0.76 (0.73–0.78) (Fig 1). The cut-off point of -0.79 predicted NAFLD with sensitivity of 69% and specificity of 71% in Chinese.

The Finns (n = 572) were more obese and had more features of the MetS than the Chinese (Table 1). In Finns, the AUROC was comparable to that in the Chinese 0.74 (0.69–0.80). The optimal cut-off defined using the Youden index was 0.68 and had a sensitivity of 79% and a specificity of 62% (Fig 1).

### Diagnostic performance of the NAFLD liver fat score, FLI and HSI in Chinese and Finns

The AUROCs of the Chinese NAFLD score and the three existing scores ranged from 0.76–0.79 in the Chinese and 0.72–0.81 in Finns. The AUROCs had overlapping 95%CI and thus did not differ significantly from each other. However, the % of subjects with NAFLD was significantly underestimated in the Chinese when determined using the NAFLD liver fat score or the FLI and cut-offs developed for these scores in Europeans (Table 4). The prevalence of NAFLD in the Chinese was 43%, 27%, 32% and 42% when calculated using the Chinese NAFLD score, NAFLD liver fat score (P<0.001), the FLI (P<0.001) and the HSI (NS, Table 4). Meanwhile, the percentage of NAFLD in the Finns also seemed to be overestimated by the Chinese NAFLD score or HSI and cut-offs originally determined in Asian populations (Table 4).

**Table 4:** Diagnostic performance of noninvasive prediction scores.

	AUROC	Cut-off (“old”)	Sensitivity (%)	Specificity (%)	% with NAFLD (“Old” cut-off)	Cut-off (“new”)	% with NAFLD (“New” cut off)
Chinese							
China NAFLD score	0.78(0.76–0.79)	-0.79	70(67–73)	73(71–75)	42.7%	-0.79	42.7%
NAFLD liver fat score	0.76(0.74–0.77)	-0.64	49(46–52)	85(83–86)	26.6%***	-1.54	42.8%
Fatty liver index	0.76(0.74–0.77)	40	57(53–60)	81(79–82)	31.6%***	27.1	45.4%
Hepatic steatosis index	0.77(0.75–0.78)	33	68(65–71)	71(70–73)	41.9%	32.5	44.7%
Finns							
China NAFLD score	0.74(0.69–0.80)	-0.79	98(95–99)	23(17–30)	87.7%***	0.68	59.0%
NAFLD liver fat score	0.81(0.77–0.86)	-0.64	86(81–91)	62(54–69)	63.2%	-0.64	63.2%
Fatty liver index	0.72(0.66–0.77)	40	85(79–90)	45(38–53)	62.5%	39	64.0%
Hepatic steatosis index	0.73(0.67–0.78)	33	97(94–99)	18(13–24)	89.7%***	40.2	61.6%

Data in parentheses are 95% confidence intervals.

***p<0.001 indicates the significance of comparing each score to the ‘China NAFLD score’ in Chinese and ‘NAFLD liver fat score’ in Finns.

### Relationship between LFAT and metabolic parameters in Chinese and Finns

**Fig 2:**
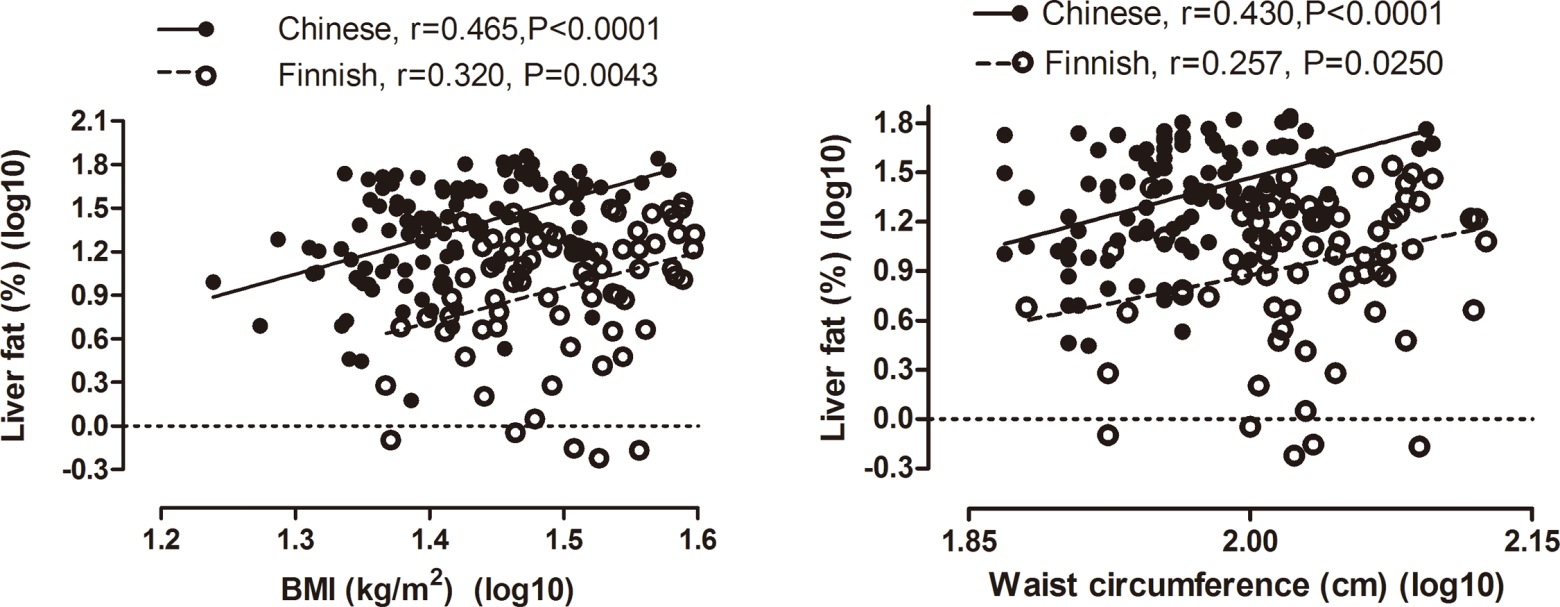
**Relationships between liver fat (%), BMI (panel on the left) and waist circumference (panel on the right) in Chinese and Finns.** There were no differences between the slopes of the regression lines relating BMI (P = 0.865) and waist circumference (P = 0.514) to ^1^H-MRS LFAT between the Chinese and Finns. The intercepts of the regression lines relating BMI (P<0.0001) and waist circumference (P<0.0001) to ^1^H-MRS LFAT were significantly higher in the Chinese than the Finns.

The regression lines relating BMI and waist circumferenceto^1^H-MRS LFAT in Chinese (n = 119) and Finns (n = 79) are shown in Fig 2. The intercepts of the regression lines relating BMI (P<0.0001) and waist circumference (P<0.0001) to ^1^H-MRS LFAT were significantly higher in the Chinese than the Finns. The slopes of the regression lines relating BMI and waist circumference to ^1^H-MRS LFAT were similar in the Chinese and the Finns. The relationship between ^1^H-MRS LFAT and fS-TG, fS-HDL, AST and ALT are shown in S2 Fig.

## Discussion

In the present study, we developed the first score for prediction of NAFLD in Han Chinese using easily available clinical and laboratory metabolic parameters, which would help clinicians to screen for patients with NAFLD. This score had comparable moderate accuracy with three previously published scores (NAFLD liver fat score, FLI and HSI) in the Chinese validation cohort and in Finns. However, the optimal cutoffs of all the NAFLD prediction scores were significantly lower in Chinese than the Finns, because for any given degree of obesity and metabolic status, the Chinese had more fat in the liver than the Finns. Thus, ethnicity needs to be considered when NAFLD is predicted using risk scores.

Knowledge of MetS and T2D, BMI, serum insulin and AST/ALT ratio allowed prediction of NAFLD with a sensitivity of 71% and a specificity of 72% in the Chinese. These parameters were similar to those previously described in Europids and Koreans [9, 12]. It has been reported previously that the major risk factors for NAFLD in China included obesity, T2DM and metabolic syndrome [22]. Mildly elevated liver enzyme concentrations, especially the serum ALT elevation relative to AST, are characteristic of NAFLD [23]. Also an increase in fasting insulin reflects hepatic insulin resistance, and previous studies have demonstrated a causal relationship between hepatic insulin resistance and liver fat content [24]. Therefore, the combination of all the above parameters constructed a prediction score for NAFLD in Chinese.

By using the Chinese NAFLD score in the Chinese community population, a Chinese NAFLD score of<-1.86 excluded NAFLD with sensitivity of 0.95, and a NAFLD index>0.49 could be used to diagnose NAFLD with specificity of 0.95. Of the 3548 subjects included in the study, 728 subjects (20.5%) had a NAFLD index<-1.86 and 461 subjects (13.0%) had a NAFLD index>0.49. This implies that approximately 30% of subjects do not need ultrasonography screening to exclude or confirm the diagnosis of NAFLD by using the Chinese NAFLD score. Therefore, the Chinese NAFLD score can be used as an efficient and simple screening tool for NAFLD in Chinese.

The Chinese NAFLD score had comparable moderate accuracy in the Chinese validation cohort and in Finns, but its cut-off value for diagnosing NAFLD was significantly lower in Chinese than the Finns. The ethnicity-dependent differences in the cut-offs for NAFLD also existed in other NAFLD prediction scores. The FLI with its cut-off developed in Europids, which includes both BMI and waist circumference, gave a significantly lower prevalence of NAFLD in the Chinese than the Chinese NAFLD score,. Also, the prevalence of NAFLD was lower in the Chinese when the NAFLD liver fat score was used. It is well known that Asians is characterized by relatively higher body fat content at lower BMI values as compared with Caucasians [25], and seems to be more prone to visceral fat accumulation [26]. In the current study, we further found that Chinese were more prone to accumulate liver fat than the Finns, at any given BMI and waist circumference. Therefore, NAFLD would occur at relatively lower value of NAFLD prediction score in Chinese than the Europids. These findings are consistent with definitions of the MetS in the Chinese, which recommend use of a lower waist circumference than in Europid subjects [26] and cross-sectional studies in Chinese, which show that the prevalence of NAFLD is the same as in Europid subjects but at a lower BMI [14]. The reason for the greater amount of liver fat in the Chinese as compared to Europids is probably due to the difference in the genes related to human body fat distribution, but it is unlikely to be the difference in the frequency of the known two most common gene variants causing NAFLD i.e. the I148M variant in PNPLA3 [27] and the E167K variant in TM6SF2 [28]. The frequency of the I148M gene variant has been reported to be 37.0% [29] and 38.0% [30] in Chinese and 37.9% in Europid subjects [31, 32]. The frequency of E167K gene variant has been reported to be 6.7% in the Chinese [33] and about 7% in Europid subjects [27,34,35].

There are several limitations in this study. Firstly, it is cross-sectional, and thus does no prove cause and effect. In a study of this size, it was not possible to obtain liver biopsies to define predictors of liver fibrosis or NASH. On the other hand, recent studies have clearly demonstrated that steatosis is an important predictor of liver fibrosis in NAFLD [36]. Secondly, since it is not possible to exchange biological samples between China and Europe, interpretation of measurements other than waist circumference and BMI i.e. fasting glucose insulin, lipids and liver enzymes should be performed with caution, although the reference ranges were very similar for these measurements in China and in Finland. Thirdly, the identification of HBV and HCV infection by medical history in Chinese population would cause missed diagnosis for some hepatitis virus carriers. Last but not the least, the Chinese NAFLD score was externally validated in a Finnish cohort with tremendous ethnicity-dependent difference in the metabolic aspects, therefore, another external validation in Asian cohort is ideally needed to further demonstrate the accuracy of Chinese NAFLD score.

We conclude that the same metabolic factors predict NAFLD in Han Chinese as in Europids. The Chinese NAFLD score can be used to predict NAFLD in both Chinese and the Europids, and previously described Europid scores such as the NAFLD liver fat score and the FLI can also be used in Chinese as well but the ethnicity-dependent cut-offs are required to ensure their diagnostic accuracy.

## Supporting Information

S1 Fig The flowchart of the study population. (TIF)

S2 Fig Relationships between liver fat (%), fS-TG (panel on the upper left), fS-HDL (panel on the upper right), fS-ALT (panel on the bottom left) and fS-AST (panel on the botton right) in Chinese and Finns. There were significant difference in the slopes of the regression lines relating fS-TG, fS-HDL, fS-ALT and fS-AST to 1H-MRS LFATbetweenthe Chinese and Finns (All P<0.05). The fS-TG, fS-HDL, fS-ALT and fS-AST were significantly higher in Finns than the Chinese at any given level of LFAT.
(JPG)

S1 Table Characteristics of the Chinese and Finnish diabetic participants. (DOCX)

## Abbreviations

ALT: alanine amino transaminase
AST: aspartate amino transaminase
BMI: body mass index
BP: blood pressure
fS: fasting serum
FLI: Fatty liver index
^1^H-MRS: proton magnetic resonance spectroscopy
HDL: high-density lipoprotein
HSI: hepatic steatosis index
LFAT: liver fat content
MetS: metabolic syndrome
NAFLD: non-alcoholic fatty liver disease
ROC: receiver operating characteristic
T2D: type 2 diabetes
TG: triglycerides
US: ultrasound
