# Supplementary figures and images for: Influence of Ethnicity on the Accuracy of Non-Invasive Scores Predicting Non-Alcoholic Fatty Liver Disease
Source: PLoS One. 2016 Aug 31;11(8):e0160526. doi: 10.1371/journal.pone.0160526 (PMC5007035; doi:10.1371/journal.pone.0160526)

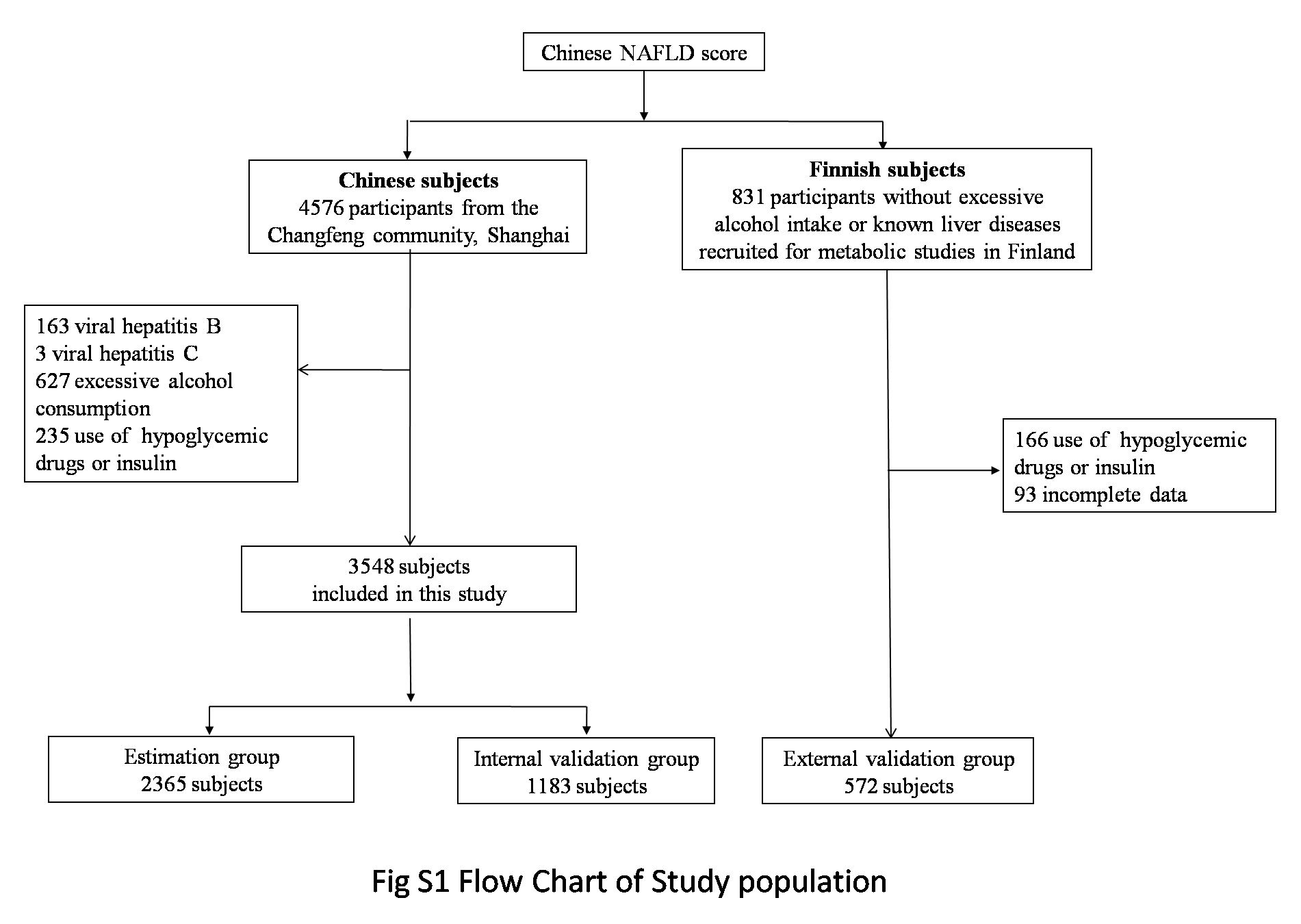

Supplement: S1 Fig — (TIF) [file pone.0160526.s001.tif]

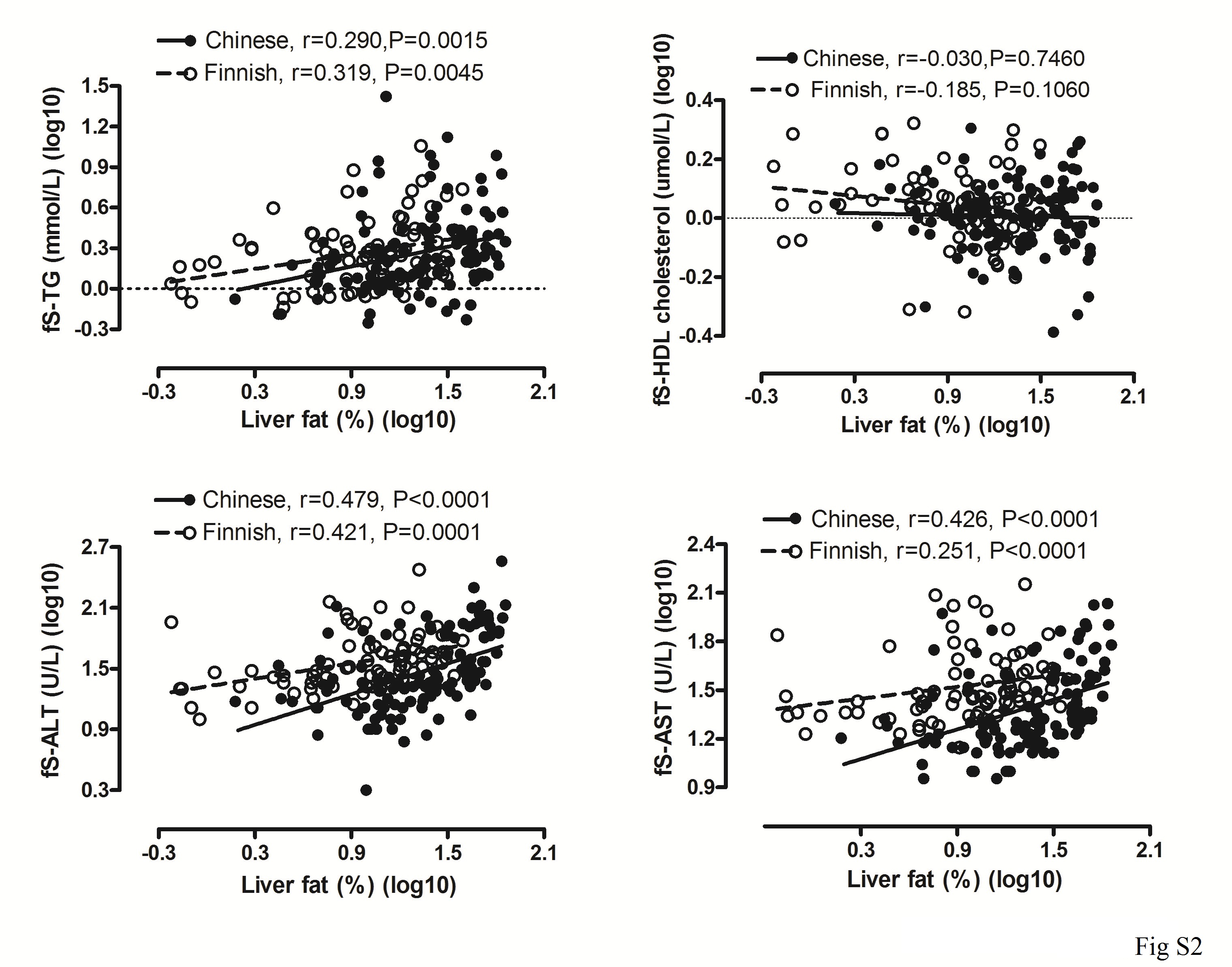

Supplement: S2 Fig — Relationships between liver fat (%), fS-TG (panel on the upper left), fS-HDL (panel on the upper right), fS-ALT (panel on the bottom left) and fS-AST (panel on the botton right) in Chinese and Finns. There were significant difference in the slopes of the regression lines relating fS-TG, fS-HDL, fS-ALT and fS-AST to 1H-MRS LFATbetweenthe Chinese and Finns (All P<0.05). The fS-TG, fS-HDL, fS-ALT and fS-AST were significantly higher in Finns than the Chinese at any given level of LFAT. (JPG) [file pone.0160526.s002.jpg]
