# Supplementary material for: Influence of Ethnicity on the Accuracy of Non-Invasive Scores Predicting Non-Alcoholic Fatty Liver Disease
Source: PLoS One. 2016 Aug 31;11(8):e0160526. doi: 10.1371/journal.pone.0160526 (PMC5007035; doi:10.1371/journal.pone.0160526)
Supplement: S1 Table — (DOCX) [file pone.0160526.s003.docx]

| Supplement Table 1 Characteristics of the Chinese and Finnish diabetic participants | | | |
| --- | --- | --- | --- |
|  | Chinese participants | Finnish participants | P value |
| N | 119 | 79 |  |
| Age(years) | 51.7±16.0 | 54.1±10.6 | 0.085 |
| Waist(cm) | 92.4±10.2 | 108.2±11.9 | <0.001 |
| BMI(kg/m2 ) | 26.1±4.0 | 32.0±4.3 | <0.001 |
| ALT (U/L) | 24(15-64) | 33(24-50) | <0.001 |
| AST (U/L) | 21(16-36) | 29(24-41) | 0.026 |
| AST/ALT ratio | 0.80(0.64-1.07) | 0.90(0.75-1.10) | 0.091 |
| fP-glucose (mmol/L) | 8.8±2.9 | 8.5±2.5 | 0.497 |
| fS-insulin (mU/L) | 10.9±7.1 | 15.4±10.7 | 0.876 |
| fS-triglycerides (mmol/L) | 1.58(1.14-2.50) | 1.72(1.24-2.54) | 0.867 |
| fS-HDL cholesterol (mmol/L) | 1.06±0.28 | 1.14±0.34 | 0.034 |
| fS-LDL cholesterol (mmol/L) | 2.8±1.0 | 2.6±0.9 | 0.186 |
| Liver fat (%) by 1H-MRS | 28.5±18.3% | 12.8±9.4% | <0.001 |
